# EEG-based analysis of various sensory stimulation effects to reduce visually induced motion sickness in virtual reality

**DOI:** 10.1038/s41598-022-21307-z

**Published:** 2022-10-27

**Authors:** Sang Seok Yeo, Jung Won Kwon, Seo Yoon Park

**Affiliations:** Department of Physical Therapy, College of Health Sciences, Dankook University, 119, Dandae-ro, Dongnam-gu, Cheonan-si, Chungnam 31116 Republic of Korea

**Keywords:** Sensory processing, Visual system

## Abstract

The use of virtual reality (VR) is frequently accompanied by motion sickness, and approaches for preventing it are not yet well established. We explored the effects of synchronized presentations of sound and motion on visually induced motion sickness (VIMS) in order to reduce VIMS. A total of 25 participants bicycle riding for 5 min with or without sound and motion synchronization presented on a head-mounted display. As a result, the VIMS scores measured by the fast motion sickness scale and simulator sickness questionnaire were significantly lower in the participants who experienced the riding scene with sound and motion than those who experienced the riding scene with sound only, motion only, or neither. Furthermore, analysis of the EEG signal showed that the higher the VIMS, the significant increase in alpha and theta waves in the parietal and occipital lobes. Therefore, we demonstrate that the simultaneous presentation of sound and motion, closely associated with synchronous and visual flow speed, is effective in reducing VIMS while experiencing simulated bicycle riding in a VR environment.

## Introduction

Virtual reality (VR) is an artificial environment created by humans using computers and other components that has since been used in a number of research^1,2^. Formerly, VR was primarily implemented on televisions, monitors, or large computer screens; however, small and lightweight head-mounted displays have recently become popular, not just in academics as well as in the public^3–5^. VR has been applied extended to various purposes, including in medicine. Various VR video content is being used including rehabilitation and clinical fields through virtual reality exposure therapy, for educational programs, video games, sports, and tourist guides^6,7^. Recently, a review to evaluate and identify research status for VR motion sickness has been reported^8^. In addition, the best-known predictive success for individual motion sickness experiences is reported to be data on quantitative kinematics of postural activity^5,9^. However, the presence of adverse effects in VR content is the main issue unresolved concern^7,10^.

Visually induced motion sickness (VIMS) is a comprehensive term that includes virtual reality motion sickness and cybersickness that is induced with a 30–80% frequency when using VR^11–13^. Although the probability and degree of VIMS are conditional on the simulator type and task, it derives from motion sickness that occurs when the human sensory organ receives conflicting inputs from the visual and vestibular systems^3,7,14^. Immersion in a virtual environment is known to cause motion sickness-like symptoms^15^. VIMS symptoms include dizziness, drowsiness, exhaustion, cold sweat, nausea, discomfort, stomach, headache, and vomiting^16,17^. Previous studies have investigated the level of VIMS utilizing various auditory and visual cues such as driving situations and VR games, however, no concord has been reached on how to reduce VIMS^18–20^. Furthermore, proposed methods for reducing VIMS (e.g., restricting the field of view) could reduce the VR experience, and immediate application of the techniques has been restricted^21,22^. Although the basic origins of VIMS remain unknown, two popular explanations in literature: sensory conflict theory and postural instability theory^22,23^. According to the sensory conflict theory, motion sickness is generated when visual, somatosensory, and vestibular signals do not correspond with a person's anticipated experiences^22,24^. The postural instability theory suggests that motion sickness is more likely to occur when an individual's mechanisms for maintaining postural stability are disrupted^25^. However, the relationship of the neural mechanisms involved in the VIMS has not yet been explained^26^.

The most commonly used qualitative evaluations are the Motion Sickness Susceptibility Questionnaire (MSSQ) and Simulator Sickness Questionnaire (SSQ) and fast motion sickness scale (FMS)^27–29^. A previous study that investigated the effects of vibration and airflow on VIMS when viewing a visual scene of driving a bicycle found a significant reduction effect of airflow on VIMS^30^. According to several studies evaluating the level of VIMS by questionnaire, simulators are reported to be caused by an inconsistency between expected and actual sensory inputs based on real experiences in terms of sensory correspondence of visual, auditory, somatosensory, and vestibular sensory information^13,22,28,29^. Therefore, presenting of more than one modality of sensory input that coincide is thought to reduce VIMS. Even though questionnaires have been frequently used for VIMS in many studies, there has been a barrier to objectively and quantitatively evaluating VIMS^11,13^.

Functional magnetic resonance imaging (fMRI), electroencephalography (EEG), electrogastrography (EGG), electrocardiography (ECG), heart rate variability (HRV), and galvanic skin response (GSR) are human body signals that could be used to measure the response to VR, including motion sickness^31^. The EEG is a method of recording the electrical activity of the brain that is used in electrophysiological monitoring^32,33^. Furthermore, because of its high accuracy and portability, EEG is one of the best methods for measuring the brain mechanics induced by motion sickness^34,35^. Several studies have found changes in EEG frequency bands for VR motion sickness^28,36–38^. Previous studies have shown that the alpha and gamma band powers of the occipital area increase as subjects increase their subjective motion sickness-related scores while performing auto-driving tasks. These results are supported by sensory conflicts the process of synthesizing signals from different sensory modalities, such as visual, vestibular, auditory, and somatosensory to produce unified percepts of the external environment^36^. Another study reported an increase the alpha and beta band power in the parietal and motor areas, as well as an increase in subjective motion sickness-related scores, using EEG analysis in a VR-based dynamic 3D environment. These areas have been associated with the integration of multiple sensory information^38^. A functional magnetic resonance imaging (fMRI) analysis of brain responses to auditory and visual associations in humans demonstrated sound-induced changes in visual motion perception. These findings show that processing auditory and visual stimuli at the same time activate the distributed nervous system in multimodal brain areas. Additionally, a direct relation was identified between subjects' perceptual experiences and activities in the cortex and subcortex^39^. It has been suggested that the occipital, parietal, and frontal areas synthesize in mediating the interaction of different-modality stimuli^39,40^. However, aspects of brain activation to multiple sensory stimuli in relation to VR motion sickness have not been investigated. Thus, using the VIMS-inducing HMD (Head Mount Display) to investigate changes in brain activity (EEG), SSQ, and FMS scores according to the level of motion sickness of an individual, objective data such as bio-signals are used to determine the individual's subjective response to motion sickness. It is necessary to quantify the level of motion sickness with support.

Therefore, this study aims to investigate the differences in VIMS according to various sensory stimuli through anatomical activation and questionnaires survey of cortical areas of the brain found in the VIMS-induced environment. In this study, we predicted that visual perception and somatosensory synchrony effectively reduced the severity of VIMS compared to unsynchronized sensory stimuli.

## Results

### Questionnaires results

**Figure 1:**
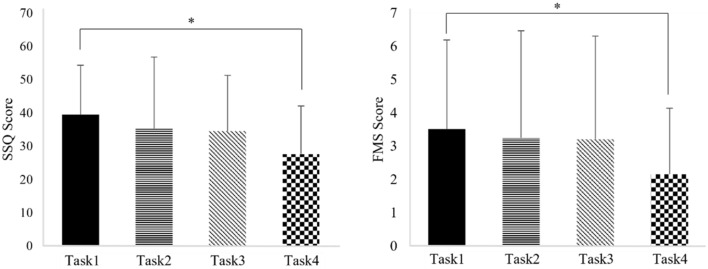
Bonferroni correction of SSQ total score and FMS score according to task performance. *SSQ* simulator sickness questionnaire, *FMS* fast motion sickness scale, *Task1* virtual reality, *Task2* virtual reality with sound, *Task3* virtual reality with riding a bicycle, *Task4* virtual reality with sound and riding a bicycle; *p < 0.05.

The SSQ and FMS score quantifies subjective feedback from the participants. One-way repeated measures ANOVA on the SSQ and FMS score for each tasks showed statistically significant differences within the tasks (p < 0.05) (Table 1). After Bonferroni correction, the SSQ and FMS score indicated a statistically significant decrease in the virtual reality with sound and riding a bicycle task compared to the VR task (p < 0.05). In contrast, there were no significant differences in SSQ and FMS scores between the VR, VR with sound, and VR with riding a bicycle tasks (p > 0.05) (Fig. 1).

**Table 1:** Comparison of the results of SSQ total score and FMS score between the tasks.

	Condition	Mean	F	p
SSQ	Task1	39.49 (14.89)	4.852	0.004*
	Task2	35.31 (21.51)		
	Task3	34.56 (16.77)		
	Task4	27.68 (14.44)		
FMS	Task1	3.51 (2.68)	3.042	0.034*
	Task2	3.25 (3.22)		
	Task3	3.21 (3.10)		
	Task4	2.16 (1.98)		

Values represent the mean (± standard deviation).

*SSQ* simulator sickness questionnaire, *FMS* fast motion sickness scale, *Task1* virtual reality, *Task2* virtual reality with sound, *Task3* virtual reality with riding a bicycle, *Task4* virtual reality with sound and riding a bicycle; *p < 0.05.

### Power changes under different conditions

**Figure 2:**
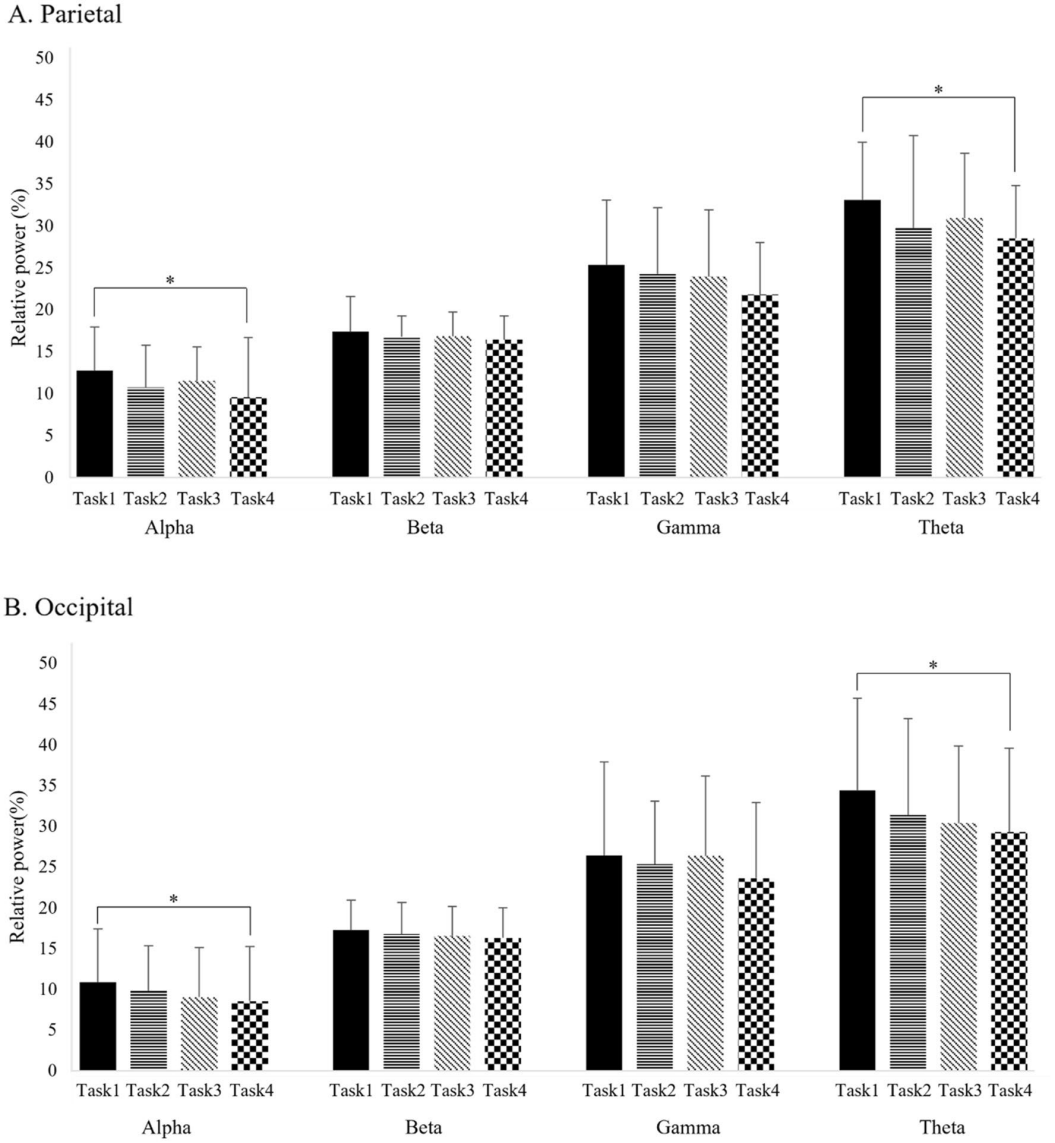
Comparison of relative power in parietal and occipital area according to task performance by Bonferroni correction. (**A**) Parietal area; (**B**) Occipital area; *Task1* virtual reality, *Task2* virtual reality with sound, *Task3* virtual reality with riding a bicycle, *Task4* virtual reality with sound and riding a bicycle; *p < 0.05.

One-way repeated measures ANOVA of relative alpha and theta power for all tasks showed statistically significant differences within the tasks (p < 0.05) (Table 2). After Bonferroni correction, the relative alpha and theta power indicated showed a statistically significant decrease in the virtual reality with sound and riding a bike task compared to the VR task (p < 0.05). In contrast, there were no significant differences in relative alpha and theta power between the VR, VR with sound, and VR with riding a bicycle tasks (p > 0.05) (Table 2). Figure 2 shows the relative power for the task condition, respectively. An alternating change in alpha and theta power of the parietal area was prominent in VR, VR with sound, VR with riding a bicycle, and VR with sound and riding a bicycle tasks (Fig. 2). An alternating change in alpha and theta power of the occipital area was prominent in VR, VR with sound, VR with riding a bicycle, and VR with sound and riding a bicycle tasks (Fig. 2).

**Table 2:** Comparison of the results of relative power in the parietal and occipital area between the tasks.

Band	Condition	Parietal				Occipital			
		Relative power	F	p	η^2^	Relative power	F	p	η^2^
Alpha	Task1	12.74 (5.22)	5.136	0.003*	0.045	10.85 (6.53)	4.526	0.006*	0.020
	Task2	10.74 (5.08)				9.78 (5.55)			
	Task3	11.53 (4.06)				9.04 (6.06)			
	Task4	9.57 (7.09)				8.53 (6.70)			
Beta	Task1	17.37 (4.20)	0.960	0.416	0.012	17.25 (3.65)	1.482	0.227	0.009
	Task2	16.79 (2.46)				16.77 (3.88)			
	Task3	16.84 (2.90)				16.55 (3.63)			
	Task4	16.42 (2.83)				16.29 (3.70)			
Gamma	Task1	25.34 (7.72)	1.826	0.150	0.030	26.41 (11.46)	0.839	0.443	0.014
	Task2	24.24 (7.94)				25.34 (7.72)			
	Task3	23.95 (7.94)				26.36 (9.79)			
	Task4	21.81 (6.20)				23.61 (9.30)			
Theta	Task1	33.12 (6.83)	2.961	0.038*	0.043	34.41 (11.29)	3.522	0.019*	0.032
	Task2	29.75 (11.02)				31.35 (11.85)			
	Task3	30.93 (7.72)				30.37 (9.48)			
	Task4	28.53 (6.30)				29.28 (10.29)			

Values represent the mean (± standard deviation).

*Task1* virtual reality, *Task2* virtual reality with sound, *Task3* virtual reality with riding a bicycle, *Task4* virtual reality with sound and riding a bicycle; *p < 0.05.

### Motion-sickness-related spectral changes

**Figure 3:**
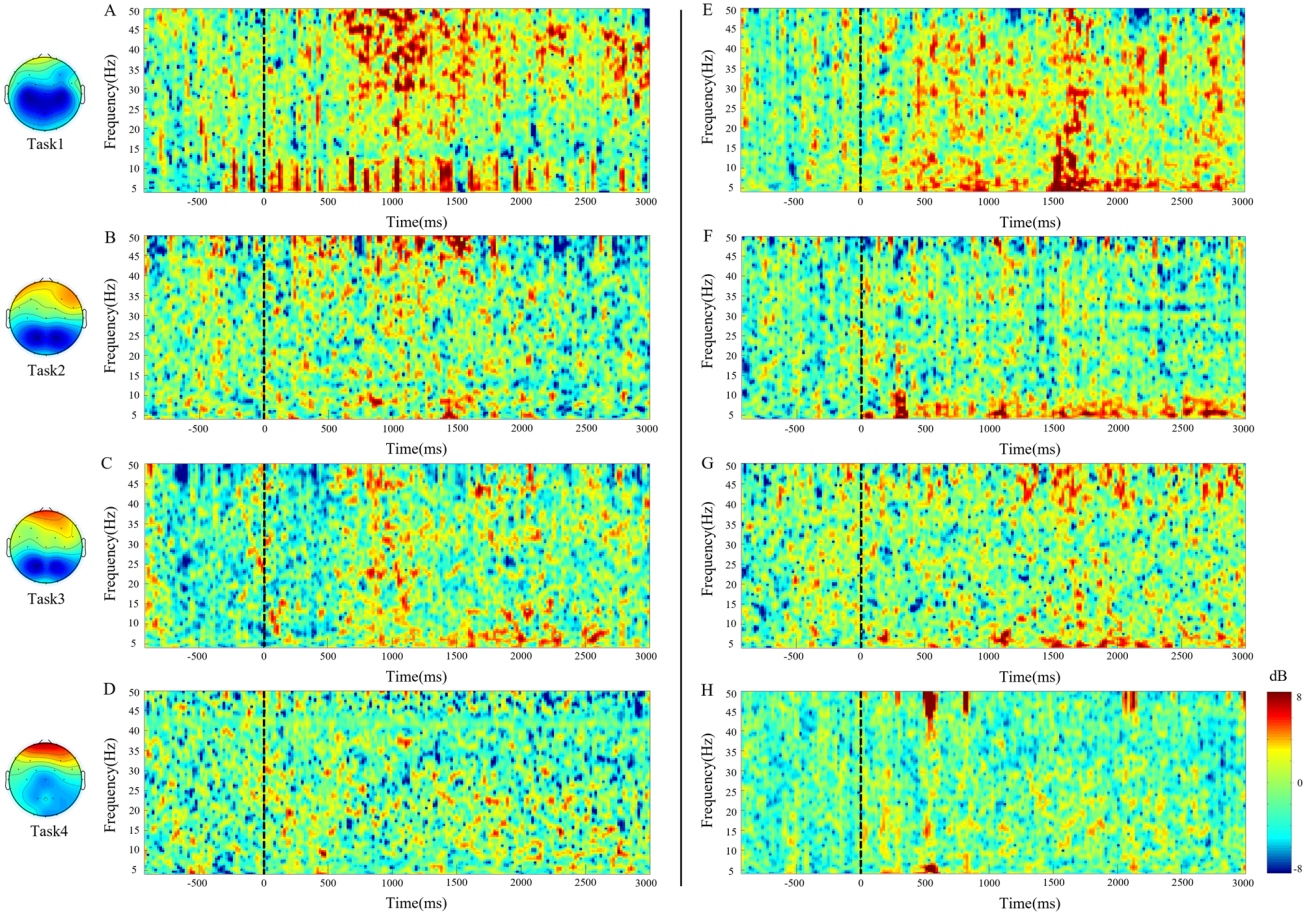
Event-related spectral perturbation analysis of the parietal and occipital area between the tasks. (**A**) Parietal virtual reality task; (**B**) Parietal virtual reality with sound task; (**C**) Parietal virtual reality with riding a bicycle task; (**D**) Parietal virtual reality with sound and riding a bicycle; (**E**) Occipital virtual reality task; (**F**) Occipital virtual reality with sound task; (**G**) Occipital virtual reality with riding a bicycle task; (**H**) Occipital virtual reality with sound and riding a bicycle; (**A**–**H**) horizontal axis: time; vertical axis: frequency; dotted line: start time of task; time–frequency response of parietal and occipital area from 25 subjects (see text).

To study the EEG correlates of motion sickness across subjects, the time–frequency responses of each task were averaged by level of motion sickness (Fig. 3). To study the EEG correlates of motion sickness across subjects, the time–frequency responses of each task were averaged by level of motion sickness (Fig. 3). The parietal and motor area (Fig. 3A,E) exhibited predominant spectral increases in all frequency bands as the motion sickness level increased. Figure 3 shows the average spectral changes in the parietal and occipital areas of 25 subjects and the brain topography during each task. ERSP responses were related to motion sickness according to task. Frequency responses at 4–8 Hz and 8–13 Hz were observed in the parietal lobe with increasing levels of motion sickness. The subjects’ dB power also shows synchronized responses in the 4–8-Hz and 8–13-Hz ranges. This motion sickness-related phenomenon is observed in parietal and occipital area.

## Discussion

In the present study, we investigated whether the synchronized presentation of sound and motion reduces motion sickness while experience a simulated bicycle ride in a VR environment, using EEG and questionnaire analyses. As a result, it showed that SSQ and FMS scores significantly decreased during the virtual reality with sound and riding a bicycle task that provided subjects with both types of additional sensory stimulation, compared to the VR task that did not provide subjects with either sound or motion stimulation. In addition, the parietal and occipital lobes exhibited significant EEG power changes in response to vestibular and visual stimuli. The ERSP of the spectral changes revealed differences in the subject's level of motion sickness during each task. During the VR task, the dB power in the alpha, beta, gamma, and theta band increased in the parietal and occipital lobe relative to the dB power recorded during the virtual reality with sound and riding a bicycle task. We found that visual perception and somatosensory are that synchronization of sensory stimulation effectively reduces the severity of VIMS compared with unsynchronized sensory stimulation.

Motion sickness felt by an individual in a VR environment could be measured using a questionnaire. The results of this study found that SSQ total scores and FMS scores were higher in the VR task and lower in the virtual reality with sound and riding a bicycle task^28–30^. Many studies have suggested that higher SSQ total and FMS scores indicate more severe motion sickness symptoms. In 2020, Sawada et al. found that when a synchronized stimulus of engine sound and vibration was applied while subjects experienced a simulated motorcycle ride in a VR environment, SSQ and FMS scores significantly decreased, and motion sickness was reduced^13^. A study that compared direct participation in a VR game to only watching showed that when subjects participated in the game, motion sickness levels were reduced. However, there was no reduction in motion sickness with sound stimulation only^41^. Several studies have shown that when motion sickness is induced, the alpha and theta power bands near the parietal lobe show the most observable variations on EEG. These results have been related to the location of the parietal lobe, a transition region between the somatosensory and motor cortex, which is involved in the integration of spatial information, including somatosensory information from vestibular sensory system input^17,28^. These studies concurrence with our results. In 2020, Li et al. compared EEG changes in HMD-based VR roaming scenes with various road conditions and found that the alpha and theta power in the parietal lobe increased as motion sickness levels increased^42^. Previous theta wave studies have concluded that the increase in parietal theta power with motion sickness is related to increased sensory input and motor planning integration^43^. It has also been suggested that theta oscillations play a role in coordinating the activity of various brain regions to update the motor plan in response to somatosensory input^44^. Therefore, increased alpha and theta power in the parietal lobe is thought to be associated with motion sickness.

Previous studies investigated the correlation between motion sickness and EEG activity in a virtual reality-based driving simulator have reported that parietal, motor, and occipital regions exhibit power changes in alpha and theta bands in response to vestibular stimulation^31,38^. In 2015, Naqvi et al. reported a significant increase in occipital lobe alpha power when the SSQ score increased. It has also been reported that increased alpha power in the parietal and occipital regions is likely to indicate the presence of motion sickness^28^. Studies on the correlation between VR symptoms and EEG recordings showed that nausea and theta power in the occipital lobe were positively correlated^31^. It has been suggested that visual information conflicts have the greatest influence and, by reducing the role of the visual domain, result in an increase in theta power. Some studies describe changes in motion sickness with increased power in the lower frequencies (delta, theta, and alpha). In particular, alpha and theta have been shown to increase in synchronization as motion sickness levels^33,42^. Certain circumstances, like as fatigue and dizziness, are associated with an increase in alpha power^45^. It must be reasoned that changes in frequency power are due to decreased vigilance by physical exertion in the VR environment. Also, changes in parietal and occipital regions are especially noticeable during stress^46^. Consequently, this change in occipital lobe power could be an indirect result of multi-sensory system conflict, increasing brain load compared to the steady-state, because the multi-sensory system conflict continues to look at the virtual environment despite motion sickness^47,48^. Motion sickness is related to the inconsistent input of motor signals transmitted by the sensory system. The vestibular apparatus has been included in the pathogenesis of motion sickness^36^. Since patients with bilateral vestibular loss do not suffer from motion sickness, it was established that the vestibular system induced motion sickness. Moreover, vestibular afferents project to various cortical areas that receive input from the visual, auditory, and somatosensory systems^4^. According to an fMRI study investigating the neural correlation between auditory input and vestibular contribution, it was reported that the region is activated for multisensory processing in the inferior and posterior insula, inferior parietal lobule, and cerebellar uvula^49^.

However, motion-sickness-induced EEG power changes are not consistent among all of the cited studies. One reason could be the different paradigms used to induce motion sickness^33,34,47^. In this study, we used a combination of visual and vestibular inputs. This could be a way to increase the realism with respect to changes in EEG power for sensory impingement in a VR environment rather than a single modality scheme. Additionally, the study performed a repeated measures within-subject design to measure VR motion sickness for multiple sensory stimuli. However, motion sickness could be changed across repeated VR exposures^15^. Therefore, consideration should be paid to the exposure interval and washout period. Nevertheless, this study has several limitations. First, it is difficult to generalize the results of this study, because the age range of the recruited subjects (20s) is rather limited, and the sample size is small. Second, since only a limited selection of sensory stimulus types was used in this study, we suggest conducting future studies that include multi-sensory feedback, such as tactile and temperature factors. Third, the presence or absence of VR experiences, and gender differences were not taken into consideration in this study. In addition, it may be difficult to determine whether physiological differences in measured EEG signal changes are caused by the emotional or physical impact of the task. However, the barrier to evaluating VIMS objectively and quantitatively arises from the fact that motion sickness is a subjective experience. Physiological and neurophysiological data generally have a weak relationship with subjective experience, so the use of objective data could not be contradicted by voluntary subjective reports. Therefore, it is important to emphasize that motion sickness is an individual problem.

## Conclusion

The present study was conducted to investigate activity changes in cerebral cortex regions and questionnaires that might be related to reductions in motion sickness-induced in a VR environment. The results obtained indicate that discrepancies between visual perception and somatosensory are associated with increases in motion sickness. Thus, provide insights that could be used in the development of VR applications that reduce motion sickness. Consequently, they suggest that the relationship between visual perception and somatosensory is important for the user to adjust to the VR environment and that synchronization of sensory stimulation is necessary.

## Methods

### Participants

Twenty-five healthy, right-handed volunteers (17 males and 8 females) with no history of cardiovascular, gastrointestinal, or vestibular diseases or drug or alcohol abuse, no medication, and normal or corrected-to-normal vision participated in this study (Table 3). All experiments were performed in accordance with relevant guidelines and regulations from the declaration of Helsinki. The measurements were conducted following the protocol approved by the Institutional Review Board (IRB) of Dankook University (DKU 2021-03-069). All participants were given a comprehensive set of instructions regarding the experiment, agreed to the experimental protocol, and provided written informed consent to participate in the study.

**Table 3:** General characteristics of the subjects.

Number of subjects	Sex (male/female)	Age (years)	Height (cm)	Weight (kg)
25	17/8	24.12 (2.80)	172.12 (8.04)	74.08 (13.38)

Values represent the mean (± standard deviation).

### Signal acquisition

**Figure 4:**
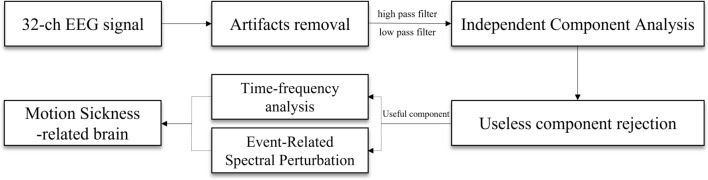
EEG signal analysis process flowchart.

Thirty-two-channel EEG signals were acquired at a sampling rate of 128 Hz using an EMOTIV EPOC Flex (Emotiv, San Francisco, CA, USA). The electrode locations were based on a 10–20 international system provided in the Matlab toolbox, EEGLAB (http://sccn.ucsd.edu/eeglab)^33,50^. The acquired EEG signals were first inspected to remove bad EEG channels. A high pass filter with a cut-off frequency at 1 Hz with a transition band of 0.2 Hz was used to remove baseline drifting and breathing artifacts. Then, a low pass filter with a cut-off frequency of 50 Hz and a transition band width of 7 Hz was applied to the signal to remove muscular artifacts and line noise^38^. Artifacts such as eye movements were removed using the EEGLAB toolbox based on Independent Component Analysis (ICA)^31,50^. Figure 4 depicts a flowchart of the procedure for EEG signal processing.

### Independent component analysis

The filtered EEG signals were decomposed into independent brain sources by ICA for biomedical time series analysis using EEGLAB^33,50^. The ICA algorithm can separate N sources from N EEG channels. The summation of the EEG sensors is assumed to be linear and instantaneous the propagation delays are negligible. We assume that the sources of muscle fission, eye, and cardiac signals are not time-locked to EEG activity, which reflects the synaptic activity of cortical neurons. As a result, the sources' time courses are assumed to be statistically independent. For EEG analysis, the rows of the input matrix X represent the EEG signals recorded at different electrodes, the rows of the output data matrix U = WX represent the time courses of activation of the ICA components, and the columns of the inverse matrix W^−1^ represent the projection strengths of the respective components onto the scalp sensors. The scalp topographies of the components reveal the location of the sources. We obtained useful components for time–frequency and ERSP analysis after removing the sources of muscle activity, eye movement, eye blinking, and single electrode noises^23,51^.

### Questionnaires (FMS and SSQ)

While each experiment, the severity of disease during EEG recordings was reported orally by each subject using FMS with a continuous scale ranging from 0 (no sickness at all) to 20 (severe sickness). It allows a quick estimate of the subject’s motion sickness level through verbal reporting^13^. Notably, the traditional SSQ was applied after each experiment to provide overall motion-sickness rating information. SSQ was composed of 16 questions that check the symptoms of motion sickness, including general discomfort, fatigue, headache, nausea, vertigo, etc.; 0 is for no symptoms, and 3 is for severe symptoms^26^. The SSQ total score ranges from 0 to 235, with higher scores indicating higher severity of symptoms.

### Time–frequency analysis

The dynamics of the ICA power spectra were tested throughout the experiment using time–frequency analysis. The power of the EEG signal was calculated as a 2-s with overlapping of 50%. The total duration of the signal was 5 min i.e., 300 s. For each participant, the absolute power was calculated for 32 electrodes, and decomposed with Fast Fourier Transform (FFT) function in MATLAB into five bands: Delta (~ 4 Hz), Theta (4–8 Hz), Alpha (8–12 Hz), Beta (12–30 Hz), and Gamma (> 30 Hz). The delta wave was excluded from the analyzes of the data collected in the current study, because it could be influenced by eye blinking and motion artifacts^52^. Two types of power were calculated from the bands, absolute and relative power. Relative power could be calculated by performing a frequency transform on the absolute power. The ratio of a band's power to the total power is referred to as the relative power. Relative power helps determine how much a given band contributes to the overall EEG^53^.

### Event related spectral perturbations (ERSP)

The time sequence of ICA activations was subject to Fast Fourier Transform (FFT) with overlapped moving windows. Spectra in each epoch were smoothed by 2-windows moving average to reduce random error. Spectra prior to event onsets were considered as baseline spectra for every trial. The mean baseline spectra were converted into dB power and subtracted from spectral power after stimulus onsets so that we can visualize spectral ‘perturbation’ from the baseline^54,55^. This procedure is applied to all the epochs, the results were then averaged to yield an ERSP image. For all cases, the continuous EEG signals were extracted into several epochs, each of which contained the sampled EEG data from − 100 to 300 s with the stimulus onset at 0 s. The ERSP image mainly showed spectral differences after the event since the baseline spectra were removed prior to event onsets. Therefore, we can compare each component by ERSP analysis of these four tasks between the tasks.

### Experimental protocol

**Figure 5:**
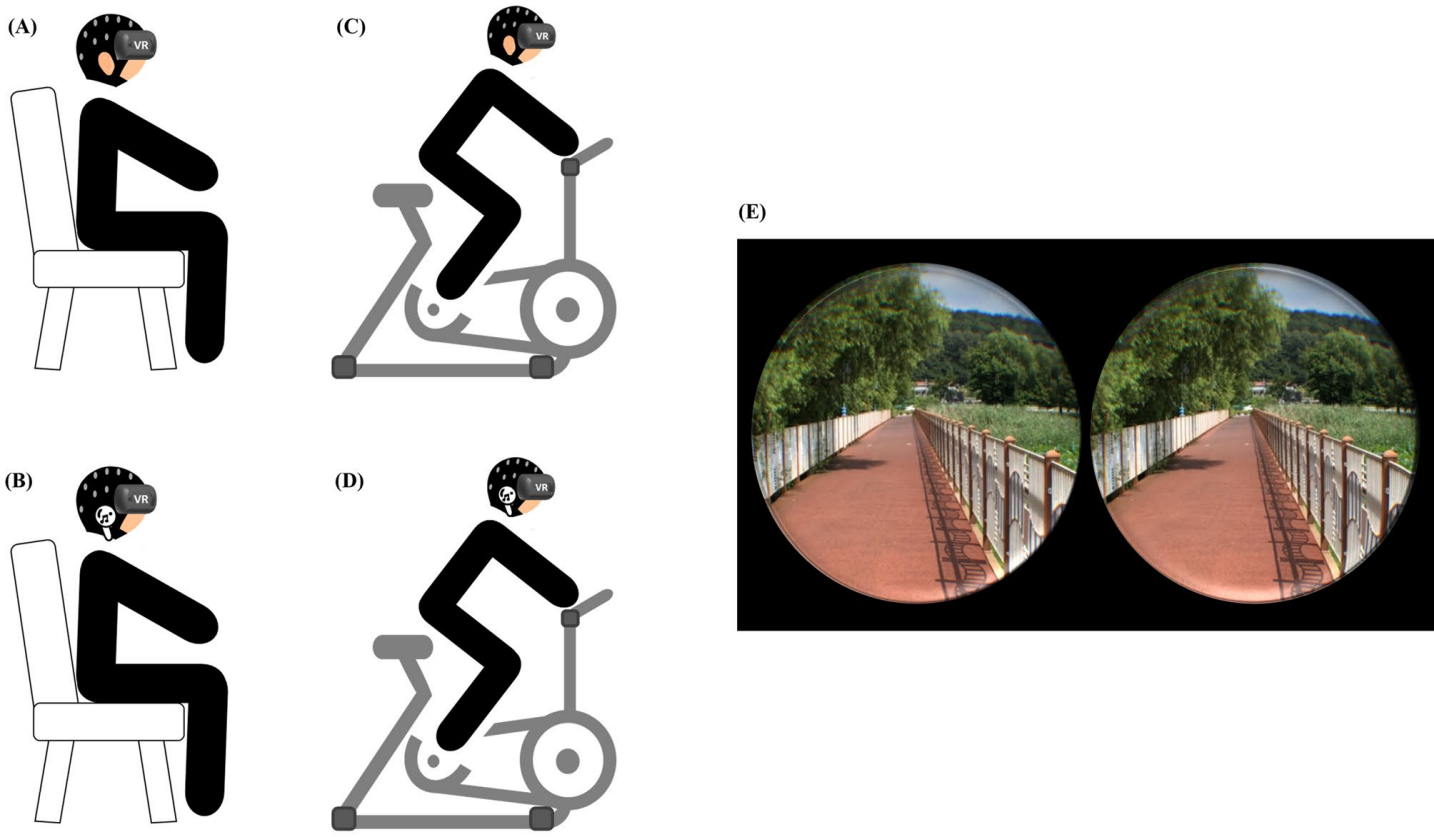
Virtual reality environment and experimental scene. (**A**) *Task1* virtual reality; (**B**) *Task2* virtual reality with sound; (**C**) *Task3* virtual reality with riding a bicycle; (**D**) *Task4* virtual reality with sound and riding a bicycle; (**E**) VR scene that simulates riding a bicycle.

For the bicycle riding HMD-VR scene, a four-task experimental protocol was designed. Before each experiment, subjects were given a 10-min explanation of the experiment and completed an SSQ questionnaire to familiarize themselves with the laboratory environment. A baseline EEG was then recorded with the EEG electrode cap and eyes closed, referred to as the “baseline phase”^32^. Then, in the bicycle riding HMD-VR phase, each of the four tasks was experienced: (1) VR task: sitting in a chair and watching a simulated bike ride through the HMD; (2) VR with sound task; sitting in a chair and watching the bike ride through the HMD while listening to the screen sounds through earphones; (3) VR with riding a bicycle task: riding a stationary bicycle while watching the bicycle ride through the HMD; and (4) VR with sound and riding a bicycle task: riding a stationary bicycle while watching the bicycle ride through the HMD and listening to the screen sounds through earphones. The VR scene comprises a 360° projection (Fig. 5). Earphones delivered sound that was coordinated with the environment while riding the bicycle while wearing the HMD. The auditory stimulus intensity in earphones for 5 min was 56-dB or 90-dB, according on the subject's selection^56^. The bicycle speed was chosen at a self-selective speed ranging from 13 to 16 km/h in kilometers per hour (km/h)^57^. Each task was experienced for 5 min, with a 10-min recovery time between tasks^15,58,59^. The task order was randomly applied. Furthermore, subjects reported their level of MS while each task verbally once per minute and completed the SSQ immediately after finishing the task^13,60^. To minimize influences on the EEG data recording, the laboratory temperature was maintained constant, and the measuring environment was quiet and free of noise.

### Statistical analysis

Data were statistically analyzed with SPSS version. 25.0. (SPSS, Inc., Chicago, IL, USA). The Shapiro–Wilk test was used to determine the normal distribution of the data of each subject. SSQ, FMS, and EEG data between the four tasks repeated were performed with one-way repeated measures ANOVA. Changes in EEG-band relative power were compared between tasks, and then the relative alpha, beta, gamma, and theta power in the parietal and occipital lobes were compared for all tasks using one-way repeated measures ANOVA. Corresponding comparisons between tasks were performed using Bonferroni correction. In the one-way repeated measures ANOVA literature, the effect size statistic is usually called eta squared (η^2^) and indicated a large effect (η^2^ = 0.14), medium effect (η^2^ = 0.06), or small effect (η^2^ = 0.01). Eta squared (η^2^) was calculated to determine the EEG-band relative power of the parietal and occipital area (Table 2). Statistical significance was accepted for p-value < 0.05.

## Data Availability

The datasets used and/or analyzed during the current study available from the corresponding author on reasonable request.

## References

[1] Ratcliffe N, Newport R (2017). The effect of visual, spatial and temporal manipulations on embodiment and action. Front. Hum. Neurosci..

[2] Roettl J, Terlutter R (2018). The same video game in 2D, 3D or virtual reality—How does technology impact game evaluation and brand placements?. PLoS One.

[3] Nooij SA (2017). Vection is the main contributor to motion sickness induced by visual yaw rotation: Implications for conflict and eye movement theories. PLoS One.

[4] Paillard AC (2013). Motion sickness susceptibility in healthy subjects and vestibular patients: Effects of gender, age and trait-anxiety. Res. Vestib. Sci..

[5] Smart LJ (2021). Simulation and virtual reality using nonlinear kinematic parameters as a means of predicting motion sickness in real-time in virtual environments. Hum. Factors..

[6] Abari, O., Bharadia, D., Duffield, A. & Katabi, D. Enabling {high-quality} untethered virtual reality. In *14th USENIX Symposium on Networked Systems Design and Implementation NSDI*, vol. 17, pp. 531–544 (2017).

[7] Saredakis D (2020). Factors associated with virtual reality sickness in head-mounted displays: A systematic review and meta-analysis. Front. Hum. Neurosci..

[8] Stanney K (2020). Identifying causes of and solutions for cybersickness in immersive technology: Reformulation of a research and development agenda. Int. J. Hum. Comput. Stud..

[9] Weech S, Varghese JP, Barnett-Cowan M (2018). Estimating the sensorimotor components of cybersickness. J. Neurophysiol..

[10] Kourtesis P, Collina S, Doumas LA, MacPherson SE (2019). Validation of the virtual reality neuroscience questionnaire: Maximum duration of immersive virtual reality sessions without the presence of pertinent adverse symptomatology. Front. Hum. Neurosci..

[11] Kourtesis P, Collina S, Doumas LA, MacPherson SE (2019). Technological competence is a pre-condition for effective implementation of virtual reality head mounted displays in human neuroscience: A technological review and meta-analysis. Front. Hum. Neurosci..

[12] Rebenitsch L, Owen C (2016). Review on cybersickness in applications and visual displays. Virtual Real..

[13] Sawada Y (2020). Effects of synchronised engine sound and vibration presentation on visually induced motion sickness. Sci. Rep..

[14] Reason JT, Brand JJ (1975). Motion Sickness.

[15] Howarth PA, Hodder SG (2008). Characteristics of habituation to motion in a virtual environment. Displays.

[16] Chen, Y. C. *et al.* Motion-sickness related brain areas and EEG power activates. In* International Conference on Foundations of Augmented Cognition* 348–354 (2009).

[17] Wei Y (2019). Motion sickness-susceptible participants exposed to coherent rotating dot patterns show excessive N2 amplitudes and impaired theta-band phase synchronization. Neuroimage.

[18] Dziuda Ł, Biernacki MP, Baran PM, Truszczyński OE (2004). The effects of simulated fog and motion on simulator sickness in a driving simulator and the duration of after-effects. Appl. Ergon..

[19] Helland A (2016). Driving simulator sickness: Impact on driving performance, influence of blood alcohol concentration, and effect of repeated simulator exposures. Accid. Anal. Prev..

[20] Munafo J, Diedrick M, Stoffregen TA (2017). The virtual reality head-mounted display Oculus Rift induces motion sickness and is sexist in its effects. Exp. Brain Res..

[21] Fernandes, A. S. & Feiner, S. K. Combating VR sickness through subtle dynamic field-of-view modification. In *2016 IEEE Symposium on 3D User Interfaces* (*3DUI*) 201–210 (2016).

[22] Oman CM (1990). Motion sickness: A synthesis and evaluation of the sensory conflict theory. Can. J. Physiol. Pharmacol..

[23] Storzer L (2016). Bicycling and walking are associated with different cortical oscillatory dynamics. Front. Hum. Neurosci..

[24] Warwick-Evans L, Symons N, Fitch T, Burrows L (1998). Evaluating sensory conflict and postural instability. Theories of motion sickness. Brain Res. Bull..

[25] Stoffregen TA, Smart LJ (1998). Postural instability precedes motion sickness. Brain Res. Bull..

[26] Lim HK (2021). Test–retest reliability of the virtual reality sickness evaluation using electroencephalography (EEG). Neurosci. Lett..

[27] Keshavarz B, Hecht H (2011). Validating an efficient method to quantify motion sickness. Hum. Factors.

[28] Naqvi SAA (2015). EEG based time and frequency dynamics analysis of visually induced motion sickness (VIMS). Australas. Phys. Eng. Sci. Med..

[29] Zhang X, Sun Y (2020). Motion sickness predictors in college students and their first experience sailing at sea. Aerosp. Med. Hum. Perform..

[30] D’Amour S, Bos JE, Keshavarz B (2017). The efficacy of airflow and seat vibration on reducing visually induced motion sickness. Exp. Brain Res..

[31] Chen Y-C (2010). Spatial and temporal EEG dynamics of motion sickness. Neuroimage.

[32] Hsin-Hung LI (2005). Study of Relationship Between Electroencephalogram Dynamics and Motion Sickness of Drivers in a Virtual Reality Dynamic Driving Environment in Hsinchu.

[33] Min BC, Chung SC, Min YK, Sakamoto K (2004). Psychophysiological evaluation of simulator sickness evoked by a graphic simulator. Appl. Ergon..

[34] Wu JP (1992). EEG changes in man during motion sickness induced by parallel swing. Space Med. Med. Eng..

[35] Chen, Y. *et al.* Assessing rTMS effects in MdDS: Cross-modal comparison between resting state EEG and fMRI connectivity. In *2017 39th Annual International Conference of the IEEE Engineering in Medicine and Biology Society *(*EMBC*) 1950–1953 (2017). 10.1109/EMBC.2017.8037231 29060275

[36] Chuang SW, Chuang CH, Yu YH, King JT, Lin CT (2016). EEG alpha and gamma modulators mediate motion sickness-related spectral responses. Int. J. Neural Syst..

[37] Lin CT, Tsai SF, Ko LW (2013). EEG-based learning system for online motion sickness level estimation in a dynamic vehicle environment. IEEE Trans. Neural Netw. Learn. Syst..

[38] Lin, C. T., Chuang, S. W., Chen, Y. C., Ko, L. W., Liang, S. F. & Jung, T. P. EEG effects of motion sickness induced in a dynamic virtual reality environment. In *2007 29th Annual International Conference of the IEEE Engineering in Medicine and Biology Society* 3872–3875 (2007). 10.1109/IEMBS.2007.4353178 18002844

[39] Bushara K (2003). Neural correlates of cross-modal binding. Nat. Neurosci..

[40] Stein BE (1998). Neural mechanisms for synthesizing sensory information and producing adaptive behaviors. Exp. Brain Res..

[41] Keshavarz B, Hecht H (2012). Visually induced motion sickness and presence in videogames: The role of sound. Proceedings of the Human Factors and Ergonomics Society Annual Meeting.

[42] Li X (2020). VR motion sickness recognition by using EEG rhythm energy ratio based on wavelet packet transform. Comput. Methods Programs Biomed..

[43] Caplan JB (2003). Human θ oscillations related to sensorimotor integration and spatial learning. J. Neurosci..

[44] Bland BH, Oddie SD (2001). Theta band oscillation and synchrony in the hippocampal formation and associated structures: The case for its role in sensorimotor integration. Behav. Brain Res..

[45] Ismail LE, Karwowski W (2020). Applications of EEG indices for the quantification of human cognitive performance: A systematic review and bibliometric analysis. PLoS One.

[46] Chen ACN, Dworkin SF, Haug J, Gehrig J (1989). Topographic brain measures of human pain and pain responsivity. Pain.

[47] Chelen WE, Kabrisky M, Rogers SK (1993). Spectral analysis of the electroencephalographic response to motion sickness. Aviat. Space Environ. Med..

[48] Heo J, Yoon G (2020). EEG studies on physical discomforts induced by virtual reality gaming. J. Electr. Eng. Technol..

[49] Oh SY, Boegle R, Ertl M, Stephan T, Dieterich M (2018). Multisensory vestibular, vestibular-auditory, and auditory network effects revealed by parametric sound pressure stimulation. Neuroimage.

[50] Delorme A, Makeig S (2004). EEGLAB: An open source toolbox for analysis of single-trial EEG dynamics including independent component analysis. J. Neurosci. Methods.

[51] Snyder KL, Kline JE, Huang HJ, Ferris DP (2015). Independent component analysis of gait-related movement artifact recorded using EEG electrodes during treadmill walking. Front. Hum. Neurosci..

[52] Jeong D, Yoo S, Jang Y (2019). Motion sickness measurement and analysis in virtual reality using deep neural networks algorithm. J. KCGS.

[53] Ko KE, Yang HC, Sim KB (2009). Emotion recognition using EEG signals with relative power values and Bayesian network. Int. J. Control Autom. Syst..

[54] Pfurtscheller G, Aranibar A (1979). Evaluation of event-related desynchronization (ERD) preceding and following voluntary self-paced movement. Electroencephalogr. Clin. Neurophysiol..

[55] Saxena, M. & Gupta, A. Exploration of temporal and spectral features of EEG signals in motor imagery tasks. In *2021 International Conference on COMmunication Systems and NETworkS* 736–740 (2021).

[56] Nakajima, S., Ino, S., Yamashita, K., Sato, M. & Kimura, A. Proposal of reduction method of Mixed Reality sickness using auditory stimuli for advanced driver assistance systems. In *2009 IEEE International Conference on Industrial Technology* 1–5 (2009).

[57] Bogacz M (2020). Comparison of cycling behavior between keyboard-controlled and instrumented bicycle experiments in virtual reality. Transp. Res. Rec..

[58] Han DU (2019). Development of a method of cybersickness evaluation with the use of 128-channel electroencephalography. Sci. Emot. Sensib..

[59] Akizuki H (2005). Effects of immersion in virtual reality on postural control. Neurosci. Lett..

[60] Lin, C. T. *et al.* Distraction-related EEG dynamics in virtual reality driving simulation. In *2008 IEEE International Symposium on Circuits and Systems* 1088–1091 (2008).

